# Clinical Screening Tools for Sarcopenia and Its Management

**DOI:** 10.1155/2016/5978523

**Published:** 2016-02-04

**Authors:** Solomon C. Y. Yu, Kareeann S. F. Khow, Agathe D. Jadczak, Renuka Visvanathan

**Affiliations:** ^1^Aged and Extended Care Services, The Queen Elizabeth Hospital, Central Adelaide Local Health Network, 28 Woodville Road, Woodville South, Adelaide, SA 5011, Australia; ^2^Adelaide Geriatrics Training and Research with Aged Care (G-TRAC) Centre, School of Medicine and Faculty of Health Science, University of Adelaide, Adelaide, SA 5000, Australia; ^3^Centre of Research Excellence: Frailty Trans-Disciplinary Research to Achieve Healthy Aging, University of Adelaide, Adelaide, SA 5000, Australia

## Abstract

Sarcopenia, an age-related decline in muscle mass and function, is affecting the older population worldwide. Sarcopenia is associated with poor health outcomes, such as falls, disability, loss of independence, and mortality; however it is potentially treatable if recognized and intervened early. Over the last two decades, there has been significant expansion of research in this area. Currently there is international recognition of a need to identify the condition early for intervention and prevention of the disastrous consequences of sarcopenia if left untreated. There are currently various screening tools proposed. As yet, there is no consensus on the best tool. Effective interventions of sarcopenia include physical exercise and nutrition supplementation. This review paper examined the screening tools and interventions for sarcopenia.

## 1. Introduction

Physiological changes to body composition with aging are well known [1]. Muscle mass is lost at a rate of approximately 8% per decade from the age of 50 years until the age of 70 years, after which weight loss is coupled with an accelerated loss of muscle mass, reaching a rate of 15% per decade [2]. There is now general agreement that sarcopenia includes loss of muscle mass, strength, and function. However, ongoing debate continues in relation to the optimal cutoff values for diagnosing sarcopenia and, more practically, the most appropriate clinical tool to use for screening [3].

Sarcopenia is common and its prevalence will rise with population aging. The prevalence of sarcopenia is said to range between 5% and 13% in community-dwelling older people aged 65 years and over. This prevalence is higher in those 80 years and older (e.g., one in five) and those who reside in residential care or in hospital setting [3]. Sarcopenia is important clinically because of the harm related to it. It has been described that sarcopenia is an independent predictor of falls [4], disability [5], loss of independence [5], and increased mortality [6–8]. In one study conducted in the United States in 2000, it was estimated that 1.5% of the total health care expenditure was attributable to sarcopenia [9]. The authors concluded that a 10% reduction in sarcopenia could potentially save US$1.1 billion in health-related costs.

According to the operational definition by European Working Group on Sarcopenia in Older People (EWGSOP), the diagnosis of sarcopenia requires the presence of low muscle mass, with either the presence of low grip strength or low physical performance [10]. Current diagnostic methods for sarcopenia include measuring muscle mass using either dual-energy X-ray absorptiometry (DXA) or bioelectrical impedance analysis (BIA) [3]. However, these tools are not practical for clinical practice because they are costly and require burdensome trips to a health facility. Other methods such as computed tomography (CT) or magnetic resonance imaging (MRI), whilst accurate, are not practical and expensive and expose patient to radiation. Therefore, using a diagnostic approach to detect presence of sarcopenia will be time consuming and expensive and requires highly specialized equipment.

Sarcopenia is very often not noticeable in earlier phases but becomes more apparent once a critical event such as a fall has occurred or disability has set in [11]. While it is possible to preserve skeletal muscle mass in older age, it is extremely challenging to regain substantial quantities once the loss has occurred. Therefore, a screening strategy to a larger population in the community that allows for early detection is important. An ideal screening test that is clinically useful should be safe, have a reasonable cutoff level defined, be cost-effective and is both valid and reliable, easily performed in clinical setting that does not require further training, with reasonable accurate sensitivity and specificity [12]. It is currently a prevalent view that screening approach is to target those who are screen “positive” or “high risk” of sarcopenia with a multidisciplinary intervention, so that prescriptive intervention by optimizing nutrition and exercise could reduce the rate of muscle loss, thus preventing sarcopenia.

This review aims to examine the literature on screening tools and interventions for sarcopenia. With this information, clinicians will hopefully be better able to make an earlier diagnosis and intervene to prevent a decline in physical health.

## 2. Screening Method for Sarcopenia

Despite increasing research into sarcopenia, there appears to be a dearth of practical and implementable clinical screening tool to support the early identification of sarcopenia in primary care. In general, current screening methods have taken the approach of either developing a screening questionnaire, a diagnostic grid, or prediction equations. Screening test is defined by sensitivity, specificity, positive predictive value (PPV), and negative predictive value (NPV). As a general rule, a “rule-out” screening test is one that has high sensitivity and high NPV, whilst a “rule-in” screening test would have high specificity and high PPV [13]. Current screening methods are mostly “rule-out” tests identifying those not at-risk of sarcopenia in the community. It is currently unknown if any one tool is superior to the others because no head-to-head comparison study has been performed to evaluate these different screening tools for sarcopenia.  Table 1 summarises all the screening tools currently available.

In 2010, the first screening method for sarcopenia was described by the EWGSOP and a two-step algorithm using gait speed assessment and handgrip strength was recommended [10]. Patients with a gait speed cutoff of ≤0.8 m/s should have their muscle mass measured to confirm presence of sarcopenia. On the other hand, those with gait speed of >0.8 m/s undergo measurement of their handgrip strength. Those with low handgrip strength will then be recommended to have muscle mass tested. The gait speed cutoff was derived from the study by Abellan van Kan et al. which found that adverse outcomes were associated with cutoff <0.8 m/s [19]. This algorithm was intended as a rule-out test. However no derivation or validation study has been reported on the development of this two-step screening algorithm. In one study of 3260 community-dwelling older people from Brazil, Mexico, and Spain, 83.4% of the total participants were suspected to have sarcopenia either by gait speed or by handgrip strength below the cutoff as suggested above [14]. Therefore this algorithm is of limited clinical utility in screening older adults for sarcopenia due to the high proportion of subjects selected to further undergo muscle mass assessment. However, this study did not establish the proportion of positively screened subjects who are actually sarcopenic. These findings indicate that the EWGSOP proposed cutoff values for gait speed and handgrip strength may not be widely usable across different populations.

Working on the principles that clinicians prefer simple questionnaires, Malmstrom and Morley developed the SARC-F where the following five domains were assessed: strength, ambulation (walking independence), rising from a chair, stair climbing, and history of falls [20]. The total score was 10 points with each domain scoring two. A score of 4 or more indicates a risk of sarcopenia and has been demonstrated to be associated with poor outcomes in older adults [20] (Table 1). This tool is intended to identify older people who require diagnostic evaluation for sarcopenia. Woo and colleagues have demonstrated the comparability of the SARC-F to three major consensus definitions for sarcopenia (American, European, and Asian) in 4000 community-dwelling older people in Hong Kong [21]. This questionnaire was found to be a suitable tool to exclude older people without sarcopenia, hence avoiding unnecessary and inconvenient investigations for those not at-risk [21]. Furthermore, Cao et al. showed that a score of SARC-F ≥ 4 is associated with poor physical function and hospitalization of falls in the previous 2 years adds to the strength and usefulness of this tool [22]. The strength of this screening tool is that the questions are very simple and it does not require complex measurements of strength or gait speed (Table 2). In addition, this tool has been linked to predicting clinical outcome and therefore has clinical relevance when the result screen is positive using this screening tool. However, the ability of this screening tool to monitor for improvement or deterioration is not known and, also, this tool has mainly been investigated in the community setting. Its efficacy in hospitals or residential aged care is not known.

Goodman et al. on the other hand have proposed a screening grid for low muscle mass by age and body mass index (BMI) [15]. The grid was derived from the National Health and Nutrition Examination Surveys (NHANES) 1999–2004 data where appendicular skeletal mass (ASM) was calculated from DXA measurements. The older person was classified as having low muscle mass if their skeletal muscle index (SMI) [ASM/height^2^] was one standard deviation (SD) below the mean SMI of young adults (20–40 years old). It should be noted that this cutoff is different to the less than two SD SMI recommended by EWSGOP to diagnose low muscle mass. This grid has been validated in a cohort of patients aged 65 years and above who attended the University of Utah Health Care System. However, this screening grid has not been externally validated or evaluated in a wider population.

Ishii et al. developed a simple screening test to identify older adults at high risk for sarcopenia based on the EWGSOP criteria of ASM, grip strength, and usual gait speed [16]. They found that the probability of sarcopenia could be estimated using a score chart, which includes three variables: age, grip strength, and calf circumference (Table 1). The sensitivity, specificity, and positive and negative predictive values of this tool are shown in Table 1 but essentially this tool worked best to rule-out those at-risk of sarcopenia. This tool was developed in a Japanese population and has not undergone external validation or been tested with other ethnic populations (Table 2).

Yu and colleagues in Australia have developed a screening method incorporating the use of an anthropometric prediction equation for appendicular skeletal muscle mass (ASM_PE_) [17, 18]. With this study, the researchers demonstrated that when the anthropometric PE was combined with a measure of muscle function such as grip strength, that screening method was able to “rule-out” those not at-risk of sarcopenia (Table 1). There is a further need to research this method with gait speed. There is also the opportunity to improve the accuracy of this method by improving the performance of the ASM_PE_.

Other anthropometric measurements such as calf circumference have been proposed as a screening tool for sarcopenia. Although calf circumference correlated with ASM in 1458 community-dwelling French women aged above 70 years, it was unable to predict sarcopenia defined by the ASM estimated with DXA [23]. Despite its low cost and ability to predict physical function, calf circumference is not a good screening tool for sarcopenia. Furthermore, this measurement has also not been studied in male participants [23].

## 3. Interventions

It is better to prevent progressive loss of skeletal muscle mass, strength, and function rather than try to restore it at older age. Preventive strategies go along with treatment interventions and should be initiated as early as possible before the loss of skeletal muscle mass, strength, and function will occur. Exercise interventions and nutritional approach play a significant role in the management of sarcopenia. The literature indicates that exercise interventions have the most significant improvement on sarcopenia [24]. Other evidence goes further to suggest that the combination of exercise and nutrition is the key intervention to prevent, treat, and slow down the progress of sarcopenia [25]. Pharmaceutical agents are still under investigation with no clear evidence of benefit yet.

## 4. Physical Activity and Exercise Intervention

Physical activity is defined as any movement produced by the contraction of skeletal muscles that increases energy expenditure [26]. The term “physical activity” comprises all kinds of activities (e.g., daily activities) while exercise is characterized as a planned, structured, and repetitive movement to improve or maintain components of physical function and fitness [27]. Therefore, exercise is a form of physical activity with a specific purpose and is typically described by type, intensity, frequency, and duration. Exercise increases muscle strength and muscle mass and improves physical performance. Evidence shows that progressive resistance and aerobic exercises are most beneficial for the prevention and treatment of sarcopenia [28].

Muscular strength is the ability to generate maximal force by a single muscle or a muscle group but this decreases with aging [10]. Muscular hypertrophy is the enhancement of muscle size through mechanical, metabolic, and hormonal processes [29]. Muscular power is a product of force and speed and it is a significant predictor of performing activities of daily living [30]. Muscular power declines more steeply with age compared to muscular strength but appears to be amenable to intervention in older people with sarcopenia [31].

### 4.1. Progressive Resistance Exercise

Resistance exercise comprises dynamic and static contractions against an external resistance with a progressive increase over time [32]. Resistant training can be executed on resistance machines in the gymnasium, by lifting weights, stretching bands, or using the individual's body weight. Resistant training improves muscle strength and mass by improving protein synthesis in skeletal muscle cells [24]. This leads to muscle hypertrophy and increases muscle power [30]. Resistance training is a safe, feasible, and effective intervention for older people and it is strongly recommended for people with sarcopenia [11, 33].

In older people, resistance exercise should be performed on two or three nonconsecutive days per week with at least one set of 8–12 repetitions (experts recommend 10–15) of the major muscle groups [34]. The load can be increased by 2–10% when two sets can be performed over the desired number on two consecutive training sessions [33].

A 2009 Cochrane review of 121 trials with 6,700 participants assessed the effects of progressive resistance training on physical function of older people [35]. In most trials, resistance exercise was performed 2-3 times a week at a high intensity. Resistance exercise had a large positive effect on muscle strength and a small but significant improvement in physical ability. There was a modest improvement in gait speed but a larger effect on getting up from a chair. The review concluded that resistance exercise is an effective intervention for improving strength and physical functioning in older people. However adverse events were not adequately reported in many studies and translation of these findings into clinical practice has to be approached cautiously.

### 4.2. Aerobic Exercise

Aerobic exercise is a form of structured physical activity using oxygen to meet the energy demands during exercise [36]. Examples of aerobic exercise are swimming, brisk walking, cycling, jogging, dancing or water aerobics. Aerobic exercise improves metabolic control, reduces oxidative stress, and optimizes exercise capacity [33]. It has also beneficial impact on sarcopenia by improving skeletal muscle insulin sensitivity; stimulating skeletal muscle hypertrophy; and increasing skeletal muscle mass [24, 37]. However, it does not produce the same magnitude of improvement in muscle mass and strength as resistance exercise, but it is still recommended for patients with sarcopenia [11, 33].  Table 3 summarises the recommendation of aerobic exercise in sarcopenic individuals.

A recent systematic review on exercise interventions for sarcopenia determined that aerobic and resistance exercise can improve muscle strength and physical function although it seems not consistently to increase muscle mass [28]. The presented recommendations illustrate a first step in the standardization of exercise interventions for sarcopenic people. However, further research is needed to determine optimal and significant exercise conditions for older sarcopenic people.

## 5. Nutritional Interventions

### 5.1. Protein Supplements

Nutrition also plays an important role in preventing and reversing sarcopenia. Daily muscle protein turnover is regulated in large part by nutrition, especially dietary protein [41]. Increasing age is associated with reduced appetite and early satiety resulting in many older people failing to meet the recommended daily dietary allowance (RDA) for protein which has important implications for skeletal muscles [11]. The current RDA in Australia for protein in an adult is 0.75 g/kg/day [42]. However, new evidence has shown that older adults will require higher dietary protein (up to 1.2 g/kg/day) to counteract age-related changes in protein metabolism and higher catabolic state associated with chronic or acute diseases [38].  Table 3 summarises the amount, type, and timing of protein ingestion. The table also included the adjusted amount of dietary protein intake in the setting of renal failure.

### 5.2. Essential Amino Acid Supplements

Branched chain amino acids (BCAA), such as leucine, at daily amount of either 2.5 g or 2.8 g in combination with resistance exercise may affect muscle protein synthesis, muscle recovery following illness, and muscle mass (Table 3) [38]. BCAA has shown beneficial effects on sarcopenic patients who are severely ill [43]. However, the number of studies using this supplement in older people is still limited and not all have shown beneficial results.

### 5.3. Beta-Hydroxy-Beta-Methylbutyrate (HMB)

HMB used alone or with combination of resistance exercise or lysine and arginine has shown some effects on improved muscle strength and physical performance in some studies (Table 3) [39, 40]. However, these studies were limited by small sample size.

### 5.4. Vitamin D

Low serum vitamin D levels (<50 nmol/L) are associated with reduced muscle strength and frailty [44]. Hence, it is paramount that a depleted serum vitamin D level be replaced and adequate intake is maintained according to current recommendations (i.e., 700 to 1000 IU/day of cholecalciferol) in all older people with sarcopenia [45]. Cholecalciferol in doses of 800 IU/day has been shown to decrease the risk of falls and this reduction is partly related to improved muscle strength [46].

## 6. Combination of Exercise and Nutrition

Regularly performed exercise, including resistance training, combined with an adequate nutritional intake seems to be the best way to prevent and treat sarcopenia [25]. There is evidence that resistance exercise combined with protein supplementation leads to greater muscle mass gain compared to resistance exercise or protein supplementation alone [47]. Other evidence suggests that the combination of exercise and amino acid supplementations can be effective in enhancing muscle strength, muscle mass, and walking speed in sarcopenia [48]. Also the dose of protein supplementation seems to play a significant role in the enhancement of resistance exercise and muscle protein synthesis. Evidence shows that resistance exercise increases muscle protein synthesis in the elderly at all protein doses, but to a greater extent with higher protein doses of 40 g [49]. Therefore, exercise and nutrition in combination should be always considered as a significant and important strategy in the prevention and treatment of sarcopenic patients.

## 7. Pharmacological Treatment

Many pharmacological agents such as myostatin inhibitor, testosterone, and angiotensin converting enzyme inhibitors and ghrelin-modulating agents are being investigated to treat sarcopenia but there is inadequate evidence to support their use in mainstream practice [50]. A recent proof-of-concept randomized-controlled phase 2 study has found that a humanized monoclonal antibody LY2495655, a myostatin inhibitor, increased lean mass and might improve functional measures of muscle power [51]. There are other pharmacological agents such as proteasome inhibitors and cyclophilin inhibitor, which are currently being evaluated in terms of their effects on skeletal muscles, but studies have so far been restricted to animal models [52, 53].

## 8. Challenges in Preventing Sarcopenia

While the idea of sarcopenia prevention and early management makes sense, the actual clinical detection and implementation of management remain a challenge, two main areas that require further research.

Firstly, a robust screening tool for sarcopenia is needed for clinical practice. Although anthropometric measurements are easy to obtain in clinical practice, their ability to predict sarcopenia is still limited. Several biological markers have been shown to be associated with skeletal muscle mass, strength, and function. However, these biomarkers may not be specific to skeletal muscle and are likely to be only weakly associated with clinically relevant outcomes [54]. For example, a recent study has found that copper and zinc ratio is associated with decline in physical function and development of disability [55]. The use of biomarkers in screening for sarcopenia requires further investigation.

Secondly, the implementation of interventions for sarcopenia comes with several challenges and barriers in older people. The awareness of the benefits of exercise and diet needs to be raised among older people. A recent systematic review confirmed that older people still believe that exercise is unnecessary or even potentially harmful [56]. Others recognize the benefits of exercise but report a range of barriers to participate in exercise interventions. Raising awareness is one of the most important strategies to enhance exercise participation among older people and to prevent sarcopenia on a long-term scale. Evidence shows that older people would be more active if they were advised to do so by their general practitioner [57].

It is more challenging for older people with activity limitations to engage in physical activity or exercises. In this situation, more targeted exercise plans will need to be designed. Another barrier in older people is the financial ability to attend exercise programs [27]. This barrier needs to be considered in planning long-term strategies to prevent and treat people with sarcopenia. From a dietary point of view, factors such as access to food, finances, and social isolation may all impact on an older person's ability to obtain optimal food intake. Furthermore many older people have difficulties with swallowing and loss of taste and smell which can lead to decrease in oral intake [11].

## 9. Conclusion

Strategies are needed to screen for sarcopenia and identify effective ways for preventive and therapeutic interventions. Several tools are currently available to screen older people for sarcopenia and further research is needed to determine which is most effective for use in the general population. Exercise and nutrition remain the cornerstone for good health and prevention of sarcopenia. Adequate dietary protein intake is an important measure to prevent or delay sarcopenia in the elderly. It is currently difficult to recommend any pharmacological agents as part of routine treatment of sarcopenia until larger long-term studies have found evidence to support their safe and effective use.

## Figures and Tables

**Table 1:** Summary of currently available screening tools for sarcopenia.

	EWGSOP algorithm [10]	SARC-F questionnaire [14]	Goodman et al. [15]	Ishii et al. [16]	Anthropometric PE [17, 18]
Description	Two-step algorithm: First step: gait speed Second step: handgrip strength if gait speed >0.8 m/s. If hand grip is low, proceed to muscle mass Muscle mass measurement if gait speed ≤0.8 m/s	Assessed 5 domains: Strength, independence walking, rising from a chair, climbing stairs, and history of falls Total score is 10 points (2 for each domain) A score of ≥4 indicates a risk for sarcopenia	Grid based on age and BMI is used to generate probability of sarcopenia which can be <0.20, 0.20–0.49 and ≥0.50	To estimate probability of sarcopenia with a score chart using age, handgrip strength, and calf circumference	PE = 10.05 + 0.35 (weight) − 0.62 (BMI) − 0.02 (age) + 5.10 (if male)

Definition of sarcopenia	EWGSOP	EWGSOP IWGS AWGS	Sarcopenia defined as low skeletal muscle index (SMI) [ASM/height^2^] <1 SD below the mean SMI of young adults (20–40 years)	EWGSOP	Sarcopenia as defined by ASM and low grip strength Men: ASM_DXA_ < 7.36 kg/m^2^ Grip strength < 30 kg Women: ASM_DXA_ 5.81 kg/m^2^ Grip strength < 20 kg

Development model	NA	Not published in a peer-reviewed journal	Development model from NHANES, USA Aged ≥ 65 years M = 3538 F = 5272	Development model from Japanese community-dwellers Aged ≥ 65 years M = 977 F = 994	Derived from healthy subject (age 18 to 83 years), Australia *n* = 195

Validation study	NA	Two studies: (a) Community dwellers in Hong Kong *n* = 4000 (b) Chinese older adults aged >65 years in various settings (i.e., community dwellers and nursing home residents) *n* = 230	Independent sample from patients in the University of Utah Health Care System, USA Aged ≥65 years M = 103 F = 103	Internal validation using bootstrapping procedure and final models were derived by correcting regression coefficient for over optimism	Independent sample from NWAHS and FAMAS community dwelling population adults ≥65 years M = 611 F = 375

Sensitivity (%)	NA	*EWGSOP* M 4.2 W 9.9 *IWGS* M 3.8 W 8.2 *AWGS* M 4.8 W 9.4	M 81.2 W 90.6	M 84.9 W 75.5	M 88.2 W 100.0

Specificity (%)	NA	*EWGSOP* M 98.7 W 94.4 *IWGS* M 99.1 W 94.6 *AWGS* M 98.8 W 94.2	M 66.2 W 66.2	M 88.2 W 92	M 95.5 W 83.0

PPV (%)	NA	*EWGSOP* M 25.8 W 14.3 *IWGS* M 54.8 W 25.2 *AWGS* M 29.0 W 8.4	M 58.5 W 54.7	M 54.4 W 72.8	M 65.2 W 29.2

NPV (%)	NA	*EWGSOP* M 90.8 W 91.8 *IWGS* M 78.4 W 82.2 *AWGS* M 91.0 W 94.4	M 86 W 94	M 97.2 W 93.0	M 98.8 W 100.0

BMI: body mass index; EWGSOP: European Working Group on Sarcopenia in Older People; IWGS: International Working Group on Sarcopenia; AWGS: Asian Working Group for Sarcopenia; FAMAS: Florey Adelaide Male Aging Study; SARC-F: slowness, independence walking, rising from chair, climbing stairs, and history of falls questionnaire; NHANES: National Health and Nutrition Examination Surveys; NWAHS: Northwestern Adelaide Healthy Study; PE: prediction equation; ASM_PE_: appendicular skeletal muscle mass as measured by anthropometric prediction equation; PPV: positive predictive value; NPV: negative predictive value; M: men; W: women.

**Table 2:** Strengths and weaknesses of sarcopenia screening tools.

	Strengths/advantages	Limitations/disadvantages
EWGSOP algorithm [10]	Simple two-step algorithm	No validation studies evaluated this tool Sensitivity, specificity, PPV, and NPV of this tool are unknown Limited clinical utility in screening older adults for sarcopenia due to the high proportion of subjects selected to further undergo muscle assessment

Goodman et al. [15]	Uses two simple variable	Age range limited to 65–85 years Adults with morbid obesity and significant disability were excluded from the derivation study Screening for only probability of low muscle mass

Ishii et al. [16]	Simple tool requiring three variables	External validity is unknown Calf circumference is not currently a routine measurement in clinical practice and therefore may require training to measure this accurately

SARC-F questionnaire [20]	Uses 5 questions without requiring measurements involving cutoff values They have comparable specificity and predictive power for adverse outcomes when validated against criteria requiring measurements developed by consensus panels (American, European, and Asian). Rapid screening and cost-effective	Low sensitivity may miss out people who are sarcopenic but classified as “not sarcopenic” according to SARC-F questionnaire not currently used in clinical practice

Anthropometric PE [17, 18]	Good discriminatory tool as a “rule-out” screening test Variables are already a routine clinical practice such as measurement of weight, height, and gender Can be used as screening tool in primary care setting	Not yet validated in care facility residents or hospital inpatients Not yet validated in non-Caucasian population.

EWGSOP: European Working Group on Sarcopenia in Older People; NPV: negative predictive value; SARC-F: slowness, assistance with walking, rising from chair, climbing stairs, and falls questionnaire; PE: prediction equation; PPV: positive predictive value.

**Table 3:** Exercise and nutritional interventions for sarcopenia.

Exercise [33]			
Type of training	Frequency	Intensity	Duration/set

Aerobic exercise	Minimum 5 days/week for moderate intensity or 3 days/week for vigorous intensity	Moderate intensity at 5-6 on a 10-point scale Vigorous intensity at 7-8 on a 10-point scale	Accumulate at least 30 min/day of moderate intensity activity in bouts of at least 10 min each continuous vigorous activity for at least 20 min/day
Resistance exercise (for major muscle groups using free weights and machines)	At least 2 days/week	Slow-to-moderate velocity 60–80% of 1 RM	8–10 exercises 1–3 sets per exercise 8–12 repetitions 1–3 min rest
Power training (to practice only after the resistance training)	Two days a week	High repetition velocity Light-to-moderate loading 30–60% of 1 RM	1–3 sets 6–10 repetitions

Nutritional supplementation [38–40]			
Intervention	Evidence or recommendation		
	Amount of protein	Type of protein	Timing

Protein supplement	At least 1.0–1.2 g/kg/day in people aged 65 years and above GFR 30–60—0.8 g/kg/day GFR <30—between 0.6 and 0.8 g/kg/day	“Fast” proteins are thought to be more beneficial compared to “slow” proteins but lacks robust evidence.	Even distribution of protein intake in main meals through the day
Vitamin D	Replace depleted serum vitamin D level and maintain adequate intake at 700 to 1000 IU/day of cholecalciferol		
^*∗*^Essential amino acid supplementation	Daily leucine 2.5 g or 2.8 g with combination of resistance exercise (benefits only shown in a small number of studies)		
^*∗*^Beta-hydroxy-beta-methylbutyrate (HMB)	HMB alone, or with combination of resistance exercise or arginine and lysine (evidence not consistently positive and only shown in a small number of studies)		

GFR: glomerular filtration rate, mL/min/1.73 m^2^. *∗*Not currently incorporated into mainstream of treatment.

## References

[1] Steen B. (1988). Body composition and aging. *Nutrition Reviews*.

[2] Grimby G., Saltin B. (1983). The ageing muscle. *Clinical Physiology*.

[3] Yu S., Umapathysivam K., Visvanathan R. (2014). Sarcopenia in older people. *International Journal of Evidence-Based Healthcare*.

[4] Landi F., Liperoti R., Russo A. (2012). Sarcopenia as a risk factor for falls in elderly individuals: results from the ilSIRENTE study. *Clinical Nutrition*.

[5] da Silva Alexandre T., de Oliveira Duarte Y. A., Ferreira Santos J. L., Wong R., Lebrao M. L. (2014). Sarcopenia according to the European working group on sarcopenia in older people (EWGSOP) versus dynapenia as a risk factor for disability in the elderly. *The Journal of Nutrition, Health & Aging*.

[6] Landi F., Liperoti R., Fusco D. (2012). Sarcopenia and mortality among older nursing home residents. *Journal of the American Medical Directors Association*.

[7] Kim J. H. E., Lim S., Choi S. H. (2014). Sarcopenia: an independent predictor of mortality in community-dwelling older Korean men. *The Journals of Gerontology Series A, Biological Sciences and Medical Sciences*.

[8] Vetrano D. L., Landi F., Volpato S. (2014). Association of sarcopenia with short- and long-term mortality in older adults admitted to acute care wards: results from the CRIME study. *The Journals of Gerontology Series A: Biological Sciences and Medical Sciences*.

[9] Janssen I., Shepard D. S., Katzmarzyk P. T., Roubenoff R. (2004). The healthcare costs of sarcopenia in the United States. *Journal of the American Geriatrics Society*.

[10] Cruz-Jentoft A. J., Baeyens J. P., Bauer J. M. (2010). Sarcopenia: European consensus on definition and diagnosis. *Age and Ageing*.

[11] Visvanathan R., Chapman I. (2010). Preventing sarcopaenia in older people. *Maturitas*.

[12] Grimes D. A., Schulz K. F. (2002). Uses and abuses of screening tests. *The Lancet*.

[13] Florkowski C. M. (2008). Sensitivity, specificity, Receiver-Operating Characteristic (ROC) curves and likelihood ratios: communicating the performance of diagnostic tests. *The Clinical Biochemist Reviews*.

[14] Lourenco R. A., Perez-Zepeda M., Gutierrez-Robledo L., Garcia-Garcia F. J., Manas L. R. (2015). Performance of the European Working Group on Sarcopenia in Older People algorithm in screening older adults for muscle mass assessment. *Age and Ageing*.

[15] Goodman M. J., Ghate S. R., Mavros P. (2013). Development of a practical screening tool to predict low muscle mass using NHANES 1999–2004. *Journal of Cachexia, Sarcopenia and Muscle*.

[16] Ishii S., Tanaka T., Shibasaki K. (2014). Development of a simple screening test for sarcopenia in older adults. *Geriatrics & Gerontology International*.

[17] Visvanathan R., Yu S., Field J. (2012). Appendicular skeletal muscle mass: development and validation of anthropometric prediction equations. *The Journal of Frailty & Aging*.

[18] Yu S., Appleton S., Chapman I. (2015). An anthropometric prediction equation for appendicular skeletal muscle mass in combination with a measure of muscle function to screen for sarcopenia in primary and aged care. *Journal of the American Medical Directors Association*.

[19] Abellan van Kan G., Cesari M., Gillette-Guyonnet S. (2013). Sarcopenia and cognitive impairment in elderly women: results from the EPIDOS cohort. *Age and Ageing*.

[20] Malmstrom T. K., Morley J. E. (2013). SARC-F: a simple questionnaire to rapidly diagnose sarcopenia. *Journal of the American Medical Directors Association*.

[21] Woo J., Leung J., Morley J. E. (2014). Validating the SARC-F: a suitable community screening tool for sarcopenia?. *Journal of the American Medical Directors Association*.

[22] Cao L., Chen S., Zou C. (2014). A pilot study of the SARC-F scale on screening sarcopenia and physical disability in the Chinese older people. *The Journal of Nutrition, Health & Aging*.

[23] Rolland Y., Lauwers-Cances V., Cournot M. (2003). Sarcopenia, calf circumference, and physical function of elderly women: a cross-sectional study. *Journal of the American Geriatrics Society*.

[24] Martone A. M., Lattanzio F., Abbatecola A. M. (2015). Treating sarcopenia in older and oldest old. *Current Pharmaceutical Design*.

[25] Deutz N. E. P., Bauer J. M., Barazzoni R. (2014). Protein intake and exercise for optimal muscle function with aging: recommendations from the ESPEN Expert Group. *Clinical Nutrition*.

[26] Montero-Fernández N., Serra-Rexach J. A. (2013). Role of exercise on sarcopenia in the elderly. *European Journal of Physical and Rehabilitation Medicine*.

[27] Freiberger E., Sieber C., Pfeifer K. (2011). Physical activity, exercise, and sarcopenia—future challenges. *Wiener Medizinische Wochenschrift*.

[28] Cruz-Jentoft A. J., Landi F., Schneider S. M. (2014). Prevalence of and interventions for sarcopenia in ageing adults: a systematic review. Report of the International Sarcopenia Initiative (EWGSOP and IWGS). *Age and Ageing*.

[29] Ratamess N. A., Alvar B. A., Evetoch T. K. (2009). American College of Sports Medicine position stand. Progression models in resistance training for healthy adults. *Medicine and Science in Sports and Exercise*.

[30] Puthoff M. L., Nielsen D. H. (2007). Relationships among impairments in lower-extremity strength and power, functional limitations, and disability in older adults. *Physical Therapy*.

[31] Metter E. J., Conwit R., Tobin J., Fozard J. L. (1997). Age-associated loss of power and strength in the upper extremities in women and men. *The Journals of Gerontology—Series A: Biological Sciences and Medical Sciences*.

[32] Phillips S. M. (2007). Resistance exercise: good for more than just Grandma and Grandpa's muscles. *Applied Physiology, Nutrition, and Metabolism*.

[33] Iolascon G., Di Pietro G., Gimigliano F. (2014). Physical exercise and sarcopenia in older people: position paper of the Italian Society of Orthopaedics and Medicine (OrtoMed). *Clinical Cases in Mineral and Bone Metabolism*.

[34] Nelson M. E., Rejeski W. J., Blair S. N. (2007). Physical activity and public health in older adults: recommendation from the American College of Sports Medicine and the American Heart Association. *Medicine & Science in Sports & Exercise*.

[35] Liu C.-J., Latham N. K. (2009). Progressive resistance strength training for improving physical function in older adults. *Cochrane Database of Systematic Reviews*.

[36] Haskell W. L., Lee I.-M., Pate R. R. (2007). Physical activity and public health: updated recommendation for adults from the American College of Sports Medicine and the American Heart Association. *Medicine and Science in Sports and Exercise*.

[37] Konopka A. R., Harber M. P. (2014). Skeletal muscle hypertrophy after aerobic exercise training. *Exercise and Sport Sciences Reviews*.

[38] Bauer J., Biolo G., Cederholm T. (2013). Evidence-based recommendations for optimal dietary protein intake in older people: a position paper from the PROT-AGE study group. *Journal of the American Medical Directors Association*.

[39] Flakoll P., Sharp R., Baier S., Levenhagen D., Carr C., Nissen S. (2004). Effect of *β*-hydroxy-*β*-methylbutyrate, arginine, and lysine supplementation on strength, functionality, body composition, and protein metabolism in elderly women. *Nutrition*.

[40] Stout J. R., Smith-Ryan A. E., Fukuda D. H. (2013). Effect of calcium *β*-hydroxy-*β*-methylbutyrate (CaHMB) with and without resistance training in men and women 65^+^yrs: a randomized, double-blind pilot trial. *Experimental Gerontology*.

[41] Rennie M. J., Wackerhage H., Spangenburg E. E., Booth F. W. (2004). Control of the size of the human muscle mass. *Annual Review of Physiology*.

[42] Truswell A., Cole-Ruthishauser I., Dresoti I., English R. (1991). *Recommended Dietary Allowance*.

[43] Biolo G., De Cicco M., Dal Mas V. (2006). Response of muscle protein and glutamine kinetics to branched-chain-enriched amino acids in intensive care patients after radical cancer surgery. *Nutrition*.

[44] Beaudart C., Buckinx F., Rabenda V. (2014). The effects of vitamin D on skeletal muscle strength, muscle mass, and muscle power: a systematic review and meta-analysis of randomized controlled trials. *The Journal of Clinical Endocrinology & Metabolism*.

[45] Morley J. E., Argiles J. M., Evans W. J. (2010). Nutritional recommendations for the management of sarcopenia. *Journal of the American Medical Directors Association*.

[46] Bischoff-Ferrari H. A., Dawson-Hughes B., Staehelin H. B. (2009). Fall prevention with supplemental and active forms of vitamin D: a meta-analysis of randomised controlled trials. *British Medical Journal*.

[47] Tieland M., Dirks M. L., van der Zwaluw N. (2012). Protein supplementation increases muscle mass gain during prolonged resistance-type exercise training in frail elderly people: a randomized, double-blind, placebo-controlled trial. *Journal of the American Medical Directors Association*.

[48] Kim H. K., Suzuki T., Saito K. (2012). Effects of exercise and amino acid supplementation on body composition and physical function in community-dwelling elderly Japanese sarcopenic women: a randomized controlled trial. *Journal of the American Geriatrics Society*.

[49] Yang Y., Breen L., Burd N. A. (2012). Resistance exercise enhances myofibrillar protein synthesis with graded intakes of whey protein in older men. *The British Journal of Nutrition*.

[50] Sakuma K., Yamaguchi A. (2012). Novel intriguing strategies attenuating to sarcopenia. *Journal of Aging Research*.

[51] Becker C., Lord S. R., Studenski S. A. (2015). Myostatin antibody (LY2495655) in older weak fallers: a proof-of-concept, randomised, phase 2 trial. *The Lancet Diabetes & Endocrinology*.

[52] Beehler B. C., Sleph P. G., Benmassaoud L., Grover G. J. (2006). Reduction of skeletal muscle atrophy by a proteasome inhibitor in a rat model of denervation. *Experimental Biology and Medicine*.

[53] Tiepolo T., Angelin A., Palma E. (2009). The cyclophilin inhibitor Debio 025 normalizes mitochondrial function, muscle apoptosis and ultrastructural defects in Col6a1^−/−^ myopathic mice. *British Journal of Pharmacology*.

[54] Cesari M., Fielding R. A., Pahor M. (2012). Biomarkers of sarcopenia in clinical trials–recommendations from the International Working Group on Sarcopenia. *Journal of Cachexia, Sarcopenia and Muscle*.

[55] Mocchegiani E., Malavolta M., Lattanzio F. (2012). Cu to Zn ratio, physical function, disability, and mortality risk in older elderly (ilSIRENTE study). *Age*.

[56] Franco M. R., Tong A., Howard K., Sherrington C., Ferreira P. H., Pinto R. Z. (2015). Older people's perspectives on participation in physical activity: a systematic review and thematic synthesis of qualitative literature. *British Journal of Sports Medicine*.

[57] Keogh J. W., Rice J., Taylor D., Kilding A. (2014). Objective benefits, participant perceptions and retention rates of a New Zealand community-based, older-adult exercise programme. *Journal of Primary Health Care*.

